# Imaging Features of Pulmonary CT in Type 2 Diabetic Patients with Multidrug-Resistant Tuberculosis

**DOI:** 10.1371/journal.pone.0152507

**Published:** 2016-03-29

**Authors:** Qisheng Song, Guoqing Zhang, Hongbo Jiang, Yanwei Ren, Xiwei Lu

**Affiliations:** 1 Department of Internal Medicine, Dalian Tuberculosis Hospital, Liaoning, China; 2 Department of Radiology, Dalian Tuberculosis Hospital, Liaoning, China; Public Health Research Institute at RBHS, UNITED STATES

## Abstract

**Background:**

Until now, radiographic manifestations of multidrug-resistant pulmonary tuberculosis (MDR- TB) in patients with diabetes mellitus (DM) have not been reported. We conducted a study to investigate the imaging features of pulmonary computed tomography (CT) for type 2 diabetic (T2DM) patients with MDR-TB.

**Methods:**

The clinical data and pulmonary CT findings of 39 type 2 diabetic patients with MDR-TB, 46 type 2 diabetic patients with drug-susceptible tuberculosis (DS-TB), and 72 pure drug-susceptible TB cases (without T2DM and MDR) treated at Dalian Tuberculosis Hospital from 2012 to 2015 were collected, and the clinical features and imaging differences of the three groups were compared.

**Results:**

The clinical characteristics of the three groups of patients were not significantly different except with respect to age and previous treatment history. However, on imaging, the patients with MDR-TB showed consolidation in and above the pulmonary segments was significantly more extensive than that seen in the DS-TB group with or without T2DM.

**Conclusion:**

Consolidation in or above multiple pulmonary segments with multiple mouth-eaten cavities and bronchial damage on pulmonary CT images in type 2 diabetic patients with tuberculosis suggests the possibility of multi-drug resistance.

## Introduction

Due to its low cure rate (only 48%) and high mortality rate [1,2,3], MDR-TB(defined as TB strains resistant to at least isoniazid and rifampin) has been a serious threat to human health. According to the "Global TB Annual Report 2014" of the World Health Organization (WHO), the incidence of MDR-TB in China is 8.32%, with 120,000–130,000 newly diagnosed cases every year [1]. Many studies have shown that patients with diabetes mellitus (DM) have an increased risk of developing MDR-TB [4–6], that is 2.1–8.8 times higher than those without DM [7,8]. However, no imaging study of diabetic patients with MDR-TB has been reported in the literature. This study retrospectively analyzed the clinical and imaging features of 39 type 2 diabetic patients with MDR-TB and without HIV or other pulmonary complications (such as COPD and other lung diseases) from 2012 to 2015. The data are expected to provide a reference for the early detection of MDR-TB in type 2 diabetic patients.

## Materials and Methods

### Ethics statement

This study was reviewed and approved by the ethics committee of Dalian Tuberculosis Hospital, all data including the records/information of the patients were analyzed anonymously and therefore no additional informed consent was required.

### Clinical information

The research was conducted in the Dalian Tuberculosis Hospital, which is a referral hospital for the management of TB patients and suspects from Dalian city, which has a population of more than six million. From January 2012 to January 2015, 1348 patients with the diagnosis of smear-positive pulmonary TB were screened. Of these, 189 patients for whom complete medical data (including clinical records, the results of drug sensitivity determinations, GeneXpert assays, and CT scans) were available for review were analyzed. Among them, 17 patients with DM and 11 pure TB cases were excluded for the reason that they also suffered from other complications such as chronic obstructive pulmonary disease (COPD), pneumonia, lung cancer, immune system disorders, or other basic disorders. The remaining 161 subjects comprised 89 DM TB patients and 72 randomly selected pure TB cases. Of the 89 DM TB patients, 43 were DM MDR/XDR-TB (resistant to any type of fluoroquinolone and at least one of the three following injectable drugs: amikacin, capreomycin or kanamycin in addition to isoniazid and rifampin); three T2DM XDR-TB patients and one type 1 DM(T1DM) TB case were excluded due to the rare number. The other 46 patients were randomly selected T2DM DS patients. All the subjects in this study were HIV-negative.

### Research methods

#### General information

The clinical data included age, gender, the time interval between the first diagnoses of DM and TB, smoking, alcohol abuse, previous TB treatment history and the glycated hemoglobin level at the first diagnosis of TB or MDR-TB.

All the cases were diagnosed as TB with sputum cultures positive. The results of GeneXpert assay for all patients with MDR-TB suggested rifampin resistance (patients with inconsistencies between GeneXpert assay results and traditional culture results were excluded).

#### CT image acquisition

All of the subjects in this study underwent CT scan when they were diagnosed. All CT examinations were performed with a GE Brightspeed 16-slice spiral CT scanner under the following scanning conditions: tube voltage of 120 kV, tube current of 100 mA, thickness of 10 mm, pitch of 1.375:1, reconstruction thickness of 7.5 mm, thickness of the axial slice reconstruction of 2.5 mm, and coronal and sagittal slice reconstruction thickness of 5 mm.

The images were examined and recorded by two radiologist and two clinical experts on MDR-TB, decisions on CT findings were reached by consensus. The imaging assessments consisted of small nodules (nodules less than 1 cm in diameter) and large nodules (nodules between 1 and 3 cm in diameter); Reticulo-nodular opacity(combined reticular and nodular opacity) and patchy opacity (including patchy shadows and irregular flaky infiltration); consolidation (including focal consolidation, segment consolidation, and lobe consolidation); the number of cavities (divided into categories consisting of one to two cavities and three or more cavities, mouth-eaten cavities were not included in the consolidation); pleural involvement (including pleural thickening or pleural effusion); mediastinal lymphadenopathy; local calcified lesions; bronchiectasis (bronchiectasis was not included in the consolidation); the number of pulmonary lobes involved; and ground-glass opacity(GGO).

### Statistical methods

SPSS 17.0 was used for data analysis. Measurement data for variables with normal distributions, such as age, were compared using t test, and those without normal distributions, such as the number of involved lobes, were compared using non-parametric tests. The count data were compared using a chi-square test, and the ranked data were compared using a non-parametric rank sum test. The imaging risk factors related to DM MDR-TB were analyzed using binary multivariate logistic regression analysis. Statistical significance was set at two-tailed *P <* 0.05.

## Results

### Characteristics of the clinical data for T2DM MDR-TB patients

The average age of T2DM MDR-TB patients was 48.67(±9.77) years, which was not significantly different (*P*>0.05) from the group of T2DM DS-TB; however, compared with the cases with pure DS-TB, it was clear that those with T2DM were older. Males were predominant in all three groups (82.1vs 89.1%vs 75.0%), with no statistically significant difference among the groups (*P*>0.05). In the MDR-TB group, the time interval between the first diagnoses of T2DM and MDR-TB was 4.74 ±(5.34) years, whereas in the DS-TB group the interval was 6.21 (±4.81) years, there was no statistically significant difference between groups (*P*>0.05). The average glycosylated hemoglobin levels measured at the first diagnosis of MDR/DS-TB were 8.94%(±1.42) and 8.92% (±1.43) in the T2DM MDR-TB and T2DM DS-TB groups, respectively, with no statistically significant difference between the groups (*P*>0.05). Obviously, previous TB treatment history was more frequent inT2DM MDR-TB patients than in DS-TB patients (48.7%vs 23.9%vs 23.6%,*P*<0.05), but there was no statistically significant difference between the groups in smoking and alcohol abuse (*P*>0.05) (Table 1, S1–S3 Tables).

**Table 1:** Comparisons of the clinical data of the three group patients.

Patient characteristics	T2DM MDR-TB^A^ (39)	T2DM DS-TB^B^ (46)	DS-TB(72) ^C^	*P* value
Age	48.67 ±9.77	52.83±10.80	42.35± 17.08	0.000^F^
Gender (male)	32(82.1%)	41(89.1%)	54 (75.0%)	0.160
Time interval^D^	4.74 ±5.34	6.21±4.81	_	0.057
Smokers vs nonsmokers	24(61.5%)	31 (67.4%)	34 (47.2%)	0.076
Alcohol abuse	4 (10.3%)	6(13.0%)	13 (18.1%)	0.505
Previoustreatment history	19(48.7%)	11 (23.9%)	17 (23.6%)	0.013^G^
HbA1C^E^(%)	8.94±1.42	8.92±1.43	_	0.382

A: Type 2 diabetic patients with multidrug-resistant tuberculosis.

B: Type 2 diabetic patients with drug-susceptible tuberculosis.

C: Pure tuberculosis patients without DM and MDR.

D: Time interval between the first diagnoses of DM and DS/MDR-TB (years).

E: Measurement of glycosylated hemoglobin at the first diagnosis of DS/MDR-TB.

F: T2DM MDR-TB = T2DM DS-TB> DS TB.

G: T2DM MDR-TB> T2DM DS-TB = DS TB.

### Comparison of imaging features in T2DM MDR-TB and DS-TB cases

Compared with the pure DS TB patients, signs of small nodules, pulmonary segment consolidation and pulmonary lobe consolidation were more frequent in T2DM TB patients(*P*<0.05). Remarkably, the signs of pulmonary lobe consolidation in T2DM MDR-TB group were far more than that in T2DM DS-TB group. Other signs including the frequency of cavities, showed no statistically significantly differences among the three groups.

Some signs, such as the reticulate opacities (0.0% vs 4.4% vs 5.6%), calcification (7.7% vs 4.4% vs 9.7%), and pericardial effusion (2.6% vs 2.2% vs 2.8%) were relatively rare (Table 2, S1–S3 Tables).

**Table 2:** Comparison of Imaging features of T2DM MDR-TB, T2DM DS-TB and pure DS-TB cases.

	T2DM MDR-TB (n/39%)	T2DM DS-TB (n/46%)	DS–TB(n/72%)	*P* value
Nodules				
Small nodules	36 (92.3)	44(95.7)	42(58,3)	0.000^E^
Large nodules	15 (38.5)	14(30.4)	26(36.1)	0.717
Reticulo-nodular opacity	0 (0.0)	2(4.4)	4(5.6)	0.337
patchy opacity	15 (38.5)	20(43.5)	27 37.5)	0.801
Interstitial involvement^A^	3 (7.7)	2 (4.4)	8(11.1)	0.438
Cavity^B^				
0 cavities	20 (51.3)	14 (30.4)	25(34.7)	
1–2 cavities	6 (15.4)	15 (32.6)	25 (34.7)	
≥3 cavities	13 (33.3)	17 (37.0)	22 (30.6)	0.393
Consolidation				
Focal consolidation	5 (12.8)	7 (15.2)	10 (13.9)	0.950
Pulmonary segment consolidation				
0 segments	25(64.1)	36 (78.3)	61(84.7)	
1 segment	6(15.4)	7 (15.2)	7(9.7)	
2 segments	6(15.4)	3 (6.5)	4 (5.6)	
≥3 segments	2 (5.1)	0(0.0)	0 (0.0)	0.023^F^
Pulmonary lobe consolidation				
0 lobes	25 (64.1)	42 (91.3)	69(95.8)	
1 lobe	10(25.6)	3 (6.5)	3(4.2)	
2 lobes	0(0.0)	1 (2.2)	0(0.0)	
≥3 lobes	4 (10.3)	0 (0.0)	0(0.0)	0.000^G^
Pleural involvement^C^	15 (38.5)	12 (26.1)	20(27.8)	0.399
Mediastinal lymphadenopathy	8 (20.5)	10 (21.7)	10(13.9)	0.472
Calcification	3 (7.7)	2 (4.4)	7(9.7)	0.563
Bronchiectasis^D^	4 (10.3)	1 (2.2)	8(11.1)	0.200
pulmonary lobes involved	3.92±1.32	3.26±1.37	3.25 ± 1.49	0.051
Pericardial effusion	1 (2.6)	1 (2.2)	2(2.8)	0.979

A: Interstitial involvement around the bronchial vascular bundle.

B: The cavity and bronchiectasis within the consolidation were not included.

C: including pleural effusion.

D: The cavity and bronchiectasis within the consolidation were not included.

E: T2DM MDR TB = T2DM DS TB>DS TB.

F: T2DM MDR TB> DS TB, T2DM MDR TB = T2DM DS TB, T2DM DS TB = DS TB.

G: T2DM MDR TB>T2DM DS TB = DS TB.

### Analysis of imaging signs related to the T2DM MDR-TB patients

In univariatebinary logistic regression analysis, there were significantly different between T2DM MDR-TB and DS-TB in terms of the presence of small nodules, cavities, pulmonary segment/lobe consolidation, pleural involvement and the number of pulmonary lobes involved. Multivariate analysis based on the univariatebinary logistic regression analysis revealed that pulmonary segment consolidation and lobe consolidation was significantly different between these two groups, with OR values of 2.64(95%CI: 1.51–4.61) and 4.49(95%CI:1.65–12.19) respectively, *P*<0.05 (Table 3, S1–S3 Tables).

**Table 3:** Binary logistic regression analysis.

	Univariate Analysis	Multivariate Analysis		
	*P* value	*P* value	OR value	95%CI^A^
Small nodules	0.019			
Large nodules	0.820			
Reticulo-nodular opacity	0.999			
patchy opacity	0.879			
Interstitial involvement	0.878			
1–2 cavities	0.032			
≥3 cavities	0.974			
Focal consolidation	0.908			
Pulmonary segment consolidation	0.004	0.001	2.64	1.51–4.61
Pulmonary lobe consolidation	0.000	0.003	4.49	1.65–12.19
Pleural involvement	0.033			
Mediastinal lymphadenopathy	0.615			
Calcification	0.989			
Bronchiectasis	0.607			
pulmonary lobes involved	0.017			
Pericardial effusion	0.733			

A: 95% confidence interval.

### Consolidation characteristics

**Fig 1:**
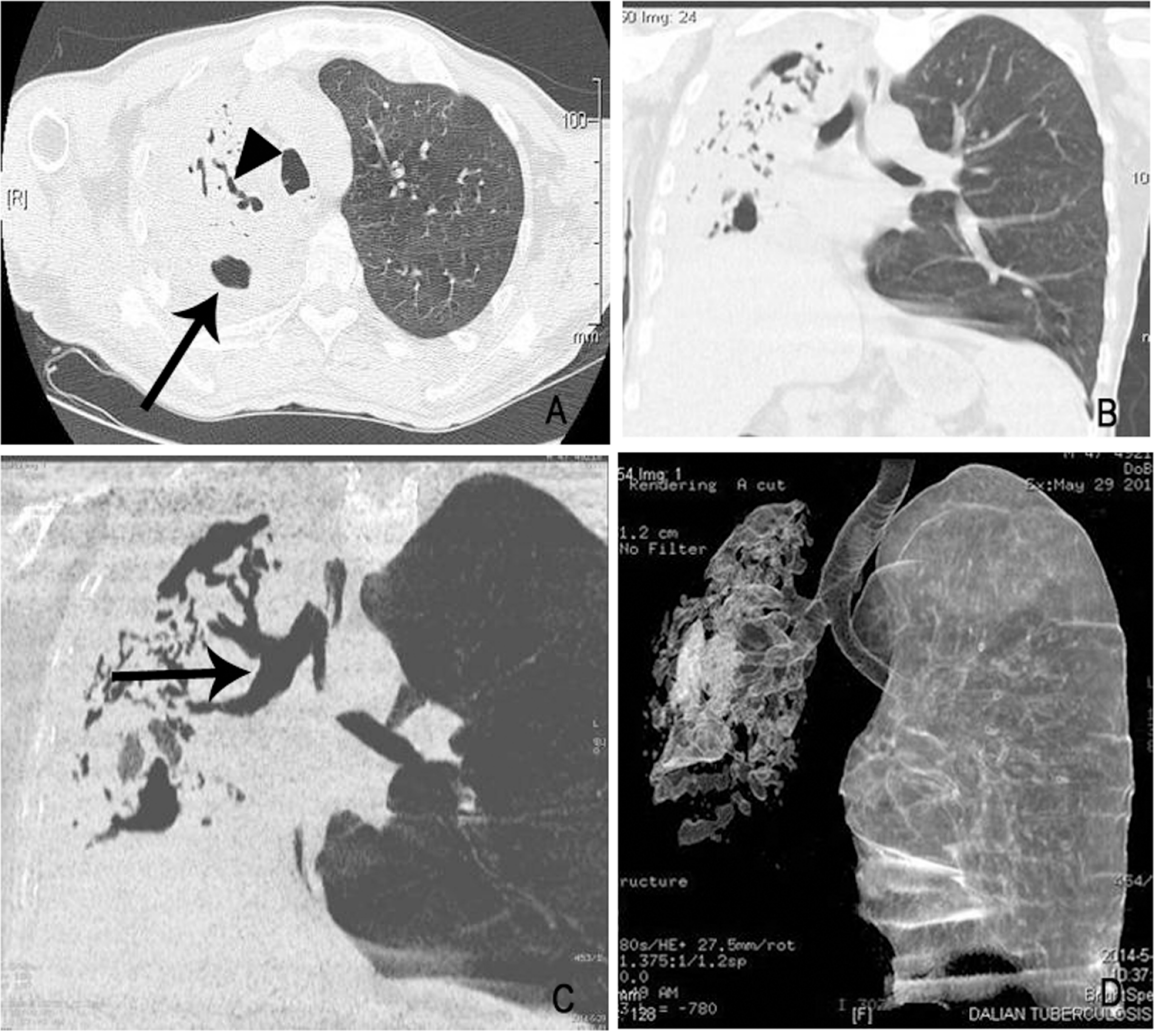
Radiological image obtained from a 47-year-old T2DM MDR-TB patient. (A) shows the volume reduction of the right upper pulmonary lobe, with mouth-eaten cavities (arrow head) and bronchial distortion (arrow) in the consolidation. (B) shows the complete consolidation of the right lung in the coronal plane of the same patient, with a mediastinal right shift. (C) shows the endobronchial varicose dilatation in the consolidation (arrow). (D): The three-dimensional reconstruction displays the destroyed right lung.

**Fig 2:**
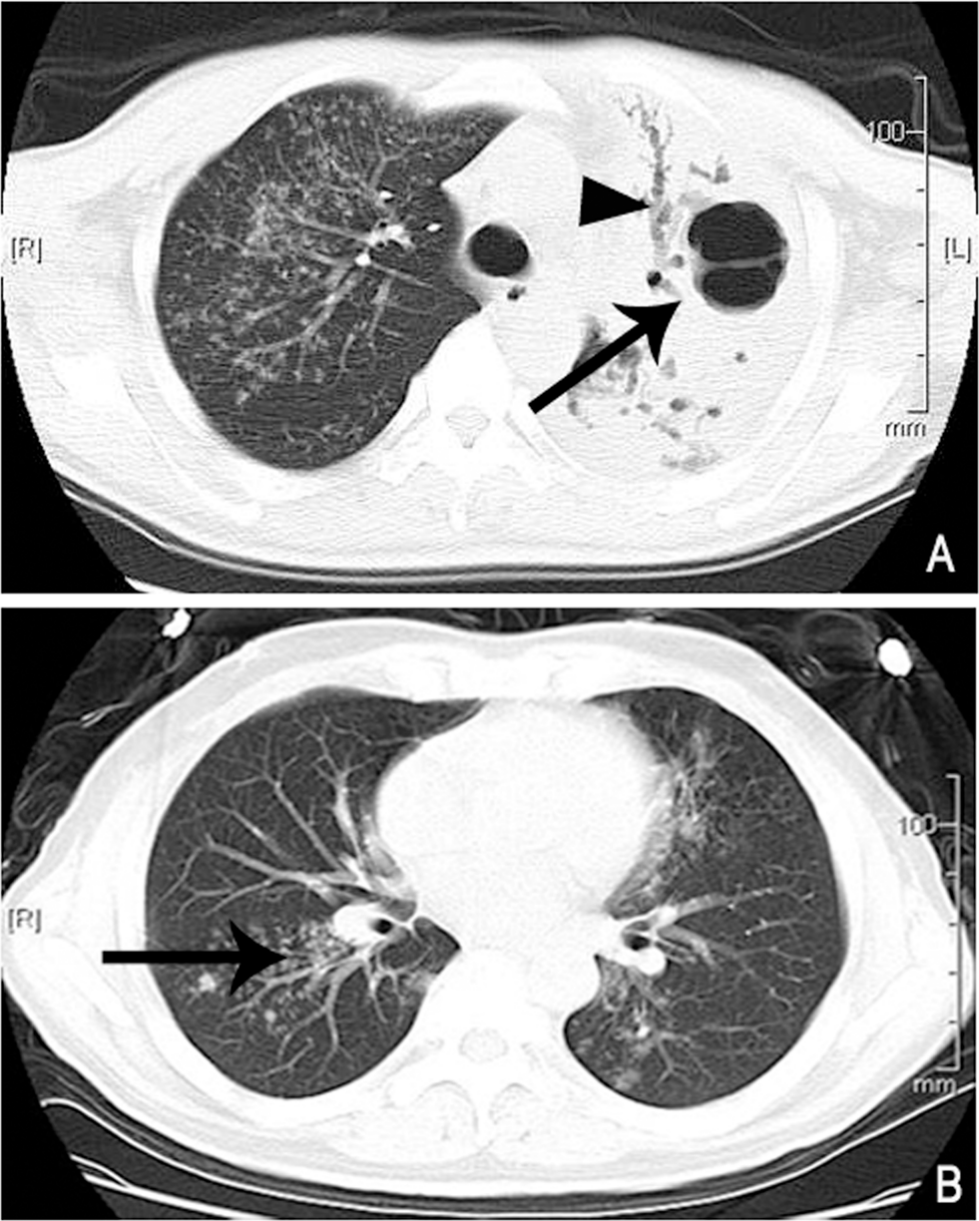
Radiological image obtained from a 51-year-old male T2DM MDR-TB patient. (A) shows the consolidation of the left upper pulmonary large lobe, with multiple mouth-eaten cavities of variable sizes (arrow) and varicose bronchiectasis in the front segment (arrow head). The right upper lobe, the inferior lingual segment of the left upper pulmonary lobe, and the basal segments of the lower lobes of both sides shown in (B), shadows of centrilobular nodules with branch lines distributed along the bronchial vascular bundle were observed, showing a tree-in-bud sign (arrow).

As indicated from the foregoing statistical analysis, pulmonary segment consolidation and lobe consolidation are risk factors associated with T2DM MDR-TB. In our observations, these consolidations (including segment and above-segment lobe consolidation) often contained multiple mouth-eaten cavities of variable size(85.7%, 24/28). Most of these cavities showed no wall or air-liquid plane, and their inner diameters ranged from 4 to 41 mm. The cavities were all located in the pulmonary segment and lobe consolidation (as shown in Figs 1 and 2), and no cavity was found in the pulmonary consolidation below the level of the sub-segment. Of the 14 cases with pulmonary segment consolidation and 14 cases with lobe consolidation, cavity formation was found in 12 cases respectively (85.7%, 12/14). In addition to cavities, the consolidations were often accompanied by significant bronchial damage. Among the 28 patients with consolidation as the main manifestation in this study, patients with bronchial morphological abnormalities in the consolidation zone, shown as a thickened bronchial wall and widened lumen in the CT scan, accounted for 85.7% (24/28) of the cases, and some cases represented as a rough inner surface of the bronchus and uneven inner wall with a lumen tortuous. Of the 24 cases with bronchial morphological abnormalities, varicose bronchiectasis accounted for 67.9% (19/28) (Figs 1 and 2); the remaining patients showed a thickened bronchial wall, widened lumen, and smooth inner membrane with normal orientation of the bronchus. Varicose bronchiectasis was found in all 14 cases with pulmonary lobe consolidation and in five cases with pulmonary segment consolidation.

**Fig 3:**
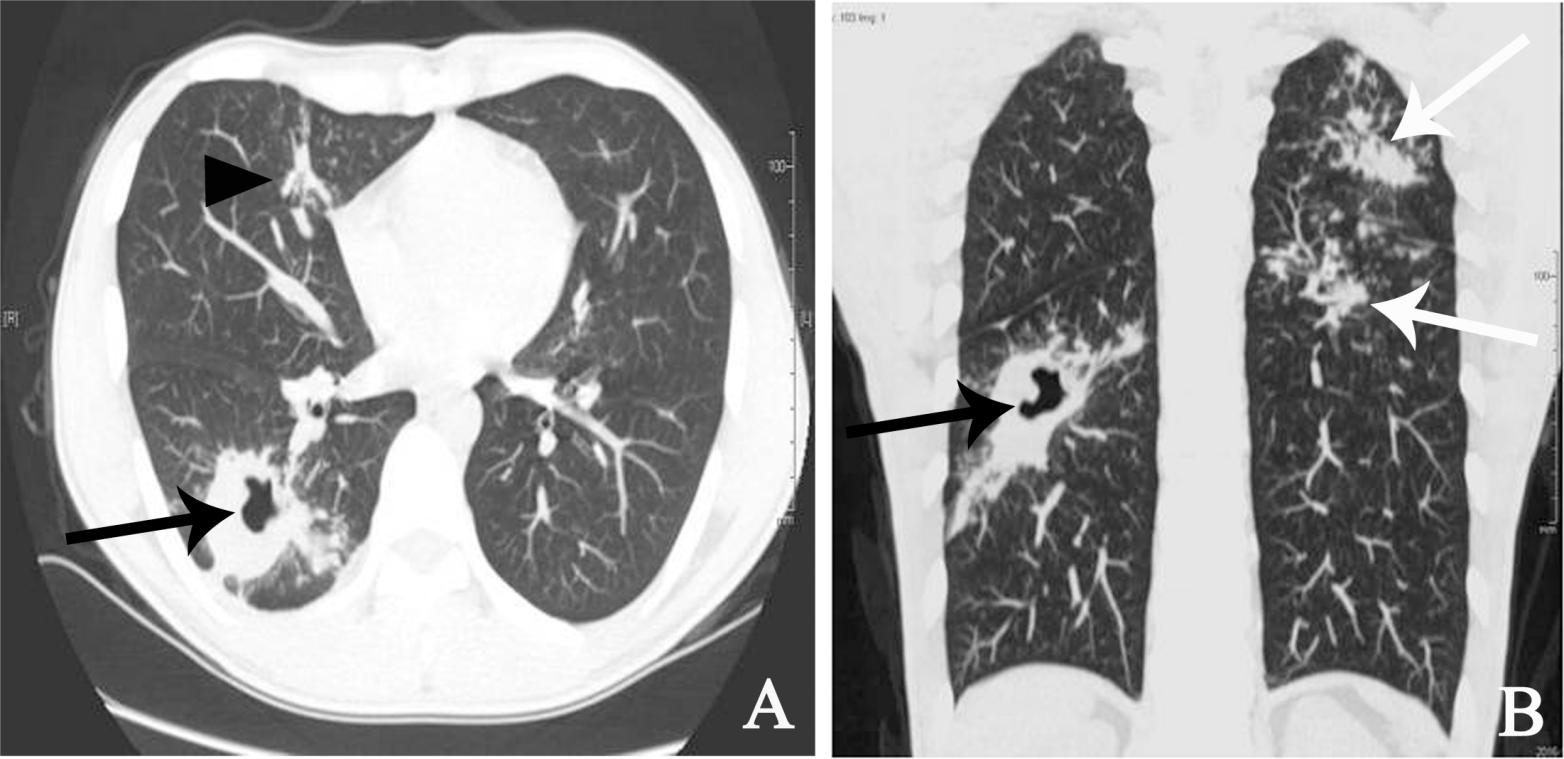
Radiological image obtained from a 28-year-old male pure DS TB patient. (A) A single thick-walled cavity located in the basal segment of the right lower lobe (black arrow) with some small nodules around it is shown; in the middle lobe of the right lung, the sign of stripe and some small centrilobular nodules can be seen (arrow head). (B). Coronal image of the same patient. In the cavity (black arrow), much small nodules were observed in the left lung, and some lesions fused into small patches in the upper lobe of the left lung were seen (white arrow).

A tree-bud sign was found around all pulmonary consolidations and the majority of the other pulmonary lobes (Fig 2). Small patchy infiltration was found around the tree-in-bud sign in six cases. Multiple thin-walled cavities were observed in the right upper lobe of one patient with full consolidation on the left side. A single thin-walled cavity was observed outside the consolidation area of the left upper lobe in one patient with multiple pulmonary segment consolidation. A high density stripe-like shadow connected to the pleura was observed at the consolidation edge in five patients with pulmonary lobe consolidation and in three patients with pulmonary segment consolidation, in addition to thickened adjacent pleura. Associated volume reduction of the pulmonary lobe or segment was found in eight patients with pulmonary segment consolidation and in six patients with pulmonary lobe consolidation (Fig 1). In contrast, in pure DS TB cases, signs of consolidation, especially the lobe consolidation, were rare (Fig 3, Table 2, S3 Table).

## Discussion

In this study, the findings regarding age are similar to those described in other published reports [9]. For both the DM MDR-TB and DM DS-TB groups, the age of the patients was relatively high, this may be due to an association of T2DM with older age. However, in terms of gender, male patients were predominant in this study, unlike other studies in which no gender difference was found. This result may be related to the different epidemic characteristics of TB in different countries and among different races [10,11]. According to the findings of this study, history of T2DM and level of glycated hemoglobin did not differ significantly in the MDR-TB and DS-TB groups, suggesting that poor blood sugar control may not be a risk factor for drug resistance in T2DM patients with TB. As reported in a previous study, we found that T2DM MDR-TB patients with previous TB treatment history were significantly more likely to have MDR TB [9].

Similar to the observation of Yeom and Fishman et al. in an imaging study of patients with primary MDR-TB[12,13], we found that consolidation, especially pulmonary lobe consolidation, is closely related to MDR-TB(P<0.01, OR: 4.49, and 95% CI: 1.65–12.19). However, Yeom et al. did not specifically describe the consolidation features of primary MDR-TB in their study. In addition, our observations in T2DM patients with MDR-TB differed from the finding of no cavities in the vast majority of consolidations that was reported by Fisherman et al [13]. The patients in our study showed the following imaging features: 1, a large consolidation range: the consolidation was mainly above the pulmonary segment, with consolidation above the pulmonary segment in 71.8% of the cases (28/39); 2, cavities with no wall associated with the consolidation: cavities with no wall mostly occurred in the consolidation area, with an incidence rate of 85.7% (12/14); 3, bronchiectasis in the consolidation area: varicose bronchiectasis often occurred, accounting for 67.9% of the cases with consolidation(19/28), and varicose bronchiectasis was found in all cases with pulmonary lobe consolidation; 4, tree-in-bud signs around the consolidation and other pulmonary lobes in all patients; and 5, volume reduction in some pulmonary lobes and segments with consolidation: volume reduction occurred in 8/14 of the cases with pulmonary segment consolidation and in 6/14 of the cases with pulmonary lobe consolidation. These different imaging findings may be related to the different strengths of the immune response between the host and the TB bacteria. Patients with severe immunodeficiency tended to manifest primary TB, whereas those with normal immune activity manifested TB in the secondary mode [14]. Perhaps limited drug penetration into the consolidation in or above multiple pulmonary segments with multiple mouth-eaten cavities and bronchial damage that harbor large numbers of mycobacteria, together with the immunodeficiency caused by the poor glucose levels control, contributed to the drug resistance.

Although some studies have suggested that the lower pulmonary lobe is more likely to be involved in DM patients with tuberculosis [15–17], the results of our study are consistent with those of other studies that do not support this finding [18,19], with no specificity found in the distribution of T2DM patients with MDR-TB and DS-TB (data not shown). At the same time, the frequency of pleural involvement (including pleural effusion) showed no significant difference between the two groups. In addition, unlike the other reports indicating that patients with MDR-TB tend to have a larger number of pulmonary lobes involved [13], we did not find the difference in this respect between the patients with T2DM MDR-TB and those with DS-TB. This might be related to the fact that relatively few cases with T2DM MDR-TB were included in this study.

Some studies have suggested that more cavities are found in patients with DM [20,21], especially in those with poor blood glucose control (HbA1c ≥ 7.0%)[22], we have the similar findings and the HbA1c values of the patients in this study were nearly all greater than 7.0%. In addition, some studies reported that the presence of multiple cavities is a risk factor for MDR-TB [23–25]. However, in our study, despite the fact that multiple cavities were common in T2DM MDR-TB patients (33.3% of the cases), statistical analysis using the Chi-square test showed no difference in the quantity or distribution of cavities in T2DM MDR-TB patients compared with T2DM DS-TB patients, nor was this parameter related to the presence of MDR-TB based on binary logistic regression analysis. A possible reason is that the number of the cavities was large and their sizes varied, especially for cavities in the consolidation, and some cavities with fusion and ill-defined boundaries were difficult to accurately count. The specific number of cavities in this study was not measured except as a categorical variable in the statistical analysis (the categories included greater than or equal to 3 cavities and less than or equal to 2 cavities). Due to the counting and statistical methods used, multiple cavities cannot be ruled out as a possible risk factor for drug resistance.

It has been reported that increased numbers of calcified lesions occur in MDR-TB patients [24,25]. In contrast, we found that the incidence of calcification was very low in T2DM patients with MDR-TB(7.7%). Although many studies on TB and calcification have been published, the specific mechanism is not yet fully understood. Studies have shown that calcification is related to iron accumulation, the transferrin receptor, and lactoferrin, as well as to the metabolism of the H-ferritin expressed by monocytes and macrophages [26]. Thus, whether the rare calcification in DM patients with TB is caused by the above mechanisms still requires further investigation.

Nodules, especially the small nodules, appeared in most of the T2DM TB patients and were far more frequent in these patients than in patients without T2DM; however, similar to findings in other research [13], binary logistic regression analysis did not indicate that this feature is related to the presence of MDR-TB.

This study has some limitations. First, the relatively small number of cases may affect the results. However, the data obtained from our imaging findings were doubly tested using a non-parametric test and a binary logistic regression test and showed consistent results; therefore, we believe that our findings indicate important reference values. Second, in the selection of DM patients with MDR-TB, some patients (n = 19) had a treatment history; therefore, we cannot determine whether the drug resistance was primary or acquired, and this factor may have had some influence on the imaging findings. However, in the patients of T2DM MDR-TB who had a treatment history, most of the sensitive mycobacterium had been killed, and the image produced by the action of the sensitive mycobacterium should have been resolved. Therefore, we believe that these imaging data might be more characteristic of drug-resistance than of mixed infections consisting of DS-TB and MDR-TB in patients who were diagnosed as MDR–TB before receiving the STR (standardized treatment regimen). Finally, because cases with T2DM XDR-TB and type 1 DM MDR/XDR-TB were rare, they were not included in this study. Assessment of differences in the imaging findings of patients with T1DM MDR-TB and DM XDR-TB will require further study.

## Conclusion

In conclusion, patients in the T2DM MDR-TB and DS-TB groups in this study showed no significant difference in clinical characteristics with the exception of previous TB treatment history. In T2DM patients with MDR-TB, calcification was rare, and the presence of multiple cavities on imaging may not be specific compared to patients without DM. Consolidation in and above multiple pulmonary segments may be a risk factor for MDR-TB development in patients with T2DM; these patients often show multiple cavities without walls in the consolidation accompanied by a high frequency of bronchial damage, particularly varicose bronchiectasis.

## Supporting Information

S1 Table Relevant data regarding the T2DMMDR-TB patients. (XLS)

S2 Table Relevant data regarding the T2DM DS-TB patients. (XLS)

S3 Table Relevant data regarding the pure TB cases without DM and MDR. (XLS)
